# Supplementary material for: Surface single-molecule dynamics controlled by entropy at low temperatures
Source: Nat Commun. 2017 Feb 9;8:14404. doi: 10.1038/ncomms14404 (PMC5309842; doi:10.1038/ncomms14404)
Supplement: Supplementary Information — Supplementary Figure and Supplementary Note [file ncomms14404-s1.pdf]

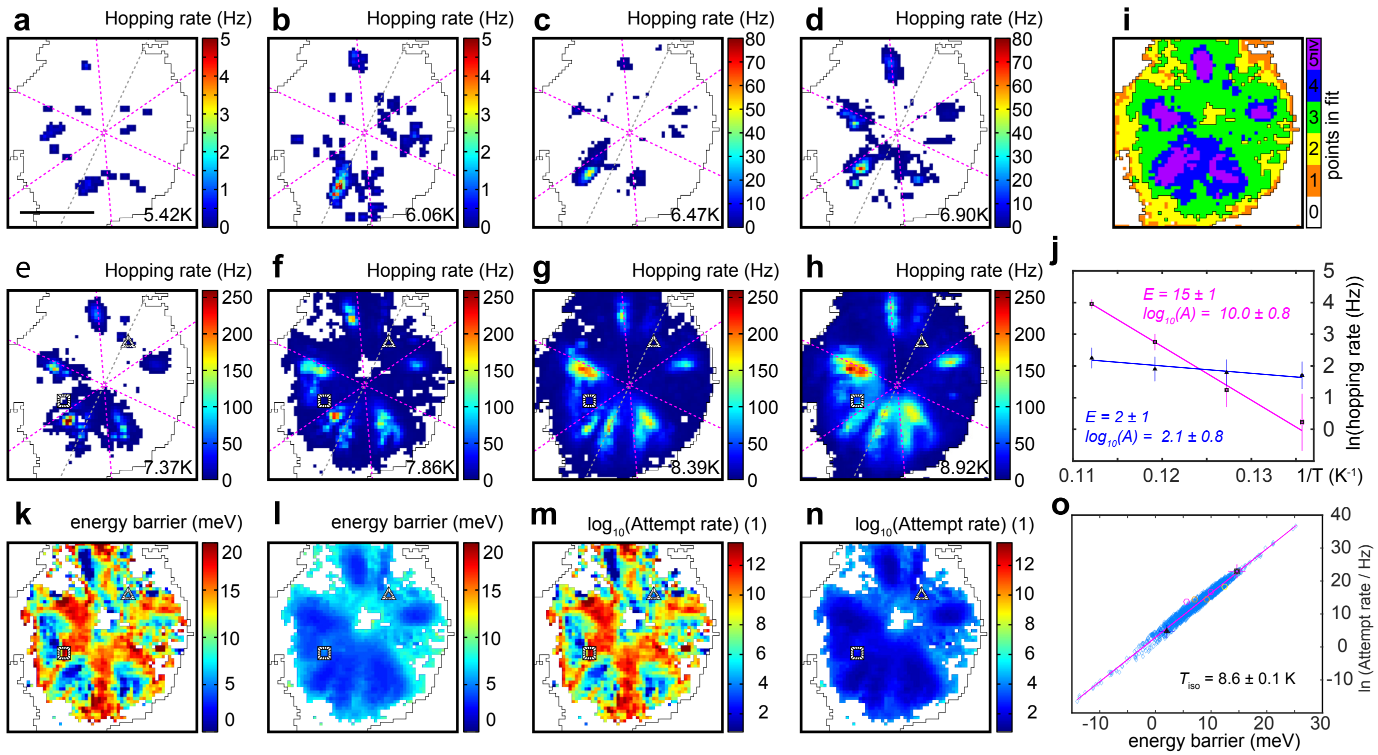


**Supplementary Figure 1. Second molecule hopping rates and analysis.** (**a – h**) Rotation-transition count of a dibutyl sulfide molecule adsorbed on the Au(111) surface at different temperatures between 5.44 K and 8.92 K. Scale bar: 1 nm. The dashed grey line is parallel to the nearby herring bone reconstruction line. The dashed magenta line comprise the perpendicular to the nearby herringbone line, and its three-fold rotation directions. (**i**) Number of rate measurements available for fitting at each relative tip-molecule position, excluding the two lowest temperatures. (**j**) Two examples of Arrhenius law fits through different positions. (**k**) Apparent energy barrier resulting from the fit for each relative tip-molecule position. (**l**) Error in the apparent energy barrier determination. (**m**) Apparent attempt rate from the fit for each relative tip-molecule position. Note the similarity of the structure displayed by the maps of (**k**) and (**m**). (**n**) Error in the apparent attempt rate. (**o**) Compensation relation between the apparent attempt rate and the apparent energy barriers for all positions, from (**k**) and (**m**) (turquoise markers; error bars omitted). The blue and magenta crosses indicate the errors for the two example points corresponding to the Arrhenius lines of Fig. (**j**). The magenta line is the linear regression of the marker data. Its slope is one over the product of the Boltzmann constant and the iso-kinetic temperature, which is thus $8.6 \pm 0.1\dot{\mathrm{K}}$. Values indicated by hexagons in yellow (fcc), magenta (fcc) and

orange (hcp), respectively, are the literature values reported in Figure 3 in the same fashion. All errors in the figure are s.d.

**Supplementary note 1**

The rate-data series obtained for a second molecule of dibutyl sulfide, shown in Supplementary Figure 1, was obtained in nearly the same conditions as reported in the main article. Exactly equivalent conditions are not possible because the STM tip changed, and we selected a molecule in a different location on the substrate. However, we carry out the measurements with the same scanning conditions otherwise. The rates depend on tip position. It is apparent that as for the first molecule, the transition rate is reaching highest levels at relative tip-molecule positions that are clustered in lobes. These lobes do not have a six-fold symmetry. As we show, there are structures of higher and lower energy barriers over the scanned area, and these match those from the attempt rate. This observation is captured also by the compensation line in Supplementary Figure 1o. It has an iso-kinetic temperature (inverse of the slope divided by the Boltzmann constant) of $8.5 \pm 0.1\,\mathrm{K}$. The isokinetic temperature is smaller than in the first molecule (Figure 3f), which we ascribe to the different molecule and location on the substrate. A slightly smaller error in this result is probably due to the different tip. As was the case in the first molecule discussed in the main text, the entropy and the enthalpy are compensated over many orders of magnitude.
